# Analysis of Recent Interception Records Reveals Frequent Transport of Arboreal Ants and Potential Predictors for Ant Invasion in Taiwan

**DOI:** 10.3390/insects11060356

**Published:** 2020-06-08

**Authors:** Ching-Chen Lee, Yi-Ming Weng, Li-Chuan Lai, Andrew V. Suarez, Wen-Jer Wu, Chung-Chi Lin, Chin-Cheng Scotty Yang

**Affiliations:** 1 Center for Ecology and Environment, Department of Life Science, Tunghai University, Taichung 40704, Taiwan; cclee86@thu.edu.tw; 2 Department of Entomology, University of Wisconsin-Madison, Madison, WI 53706, USA; weng22@wisc.edu; 3 Department of Ecological Humanities, Providence University, Taichung 43301, Taiwan; lclai@pu.edu.tw; 4 Department of Entomology, University of Illinois, Urbana-Champaign, Urbana, IL 61801, USA; suarez2@illinois.edu; 5 Department of Evolution, Ecology, and Behavior, University of Illinois, Urbana-Champaign, Urbana, IL 61801, USA; 6 Beckman Institute for Science and Technology, Urbana-Champaign, Urbana, IL 61801, USA; 7 Department of Entomology, National Taiwan University, Taipei 10617, Taiwan; wuwj@ntu.edu.tw; 8 Department of Biology, National Changhua University of Education, Changhua 50007, Taiwan; cclin@cc.ncue.edu.tw; 9 Research Institute for Sustainable Humanosphere, Kyoto University, Kyoto 611-0011, Japan; 10 Department of Entomology, Virginia Polytechnic Institute and State University, Blacksburg, VA 24061, USA; 11 Department of Entomology, National Chung Hsing University, Taichung 402204, Taiwan

**Keywords:** commodity, exotic ant, introduction pathway, life-history trait, propagule pressure, risk assessment, secondary introductions

## Abstract

We uncovered taxonomic diversity, country of origin and commodity type of intercepted ants at Taiwanese borders based on an 8 year database of 439 interception records. We found intercepted ants arrived predominantly via timber, a pattern likely reflecting the high domestic demand for foreign timber in Taiwan. The most frequently intercepted species were either arboreal or wood-dwelling ants, raising a concern of these ants constituting a next wave of ant invasion in Taiwan. Further analyses indicate that the taxonomic composition of intercepted ants does not match that of established non-native ant species, suggesting that interception data alone fails to provide adequate power to predict the establishment success of ants. Yet, interception frequency and selected life-history traits (i.e., flexible colony founding mode and general nesting habits) were shown to jointly serve as a practical predictor of the establishment risk of non-native ants. Consistent with other border interception databases, secondary introduction (i.e., species arriving from their introduced ranges instead of their native ranges) also represents a major pathway for transport of invasive ants into Taiwan, suggesting its role in shaping the global invasion of ants. Our findings offer baseline information for constructing a prediction framework for future ant invasions and assist in the decision-making process of quarantine authorities in Taiwan.

## 1. Introduction

Global trade has facilitated the movement of organisms across geographical barriers, allowing species to be transported into regions beyond their natural range [1,2]. Once established, introduced species may continue to spread and pose threats to the native fauna and flora, the economy, and even human health [3,4,5]. Moreover, they are difficult or often impossible to eradicate from invaded environments. Hence, risk assessment, along with relevant biosecurity measures, has been advocated as an effective way to prevent invasive species from entering borders [6,7]. Risk assessment also provides scientific evidence to inform proper quarantine measures, which is essential when management can incur considerable costs.

Invasive ants are commonly transported as hitchhikers or stowaways on human commerce [8,9,10]. Their long-distance spread also is frequently associated with human transportation [9,10,11,12], highlighting the importance of incorporating pathway analysis in a risk assessment framework for invasive ants. Analyses of ant interception records can also reveal the relative role of transport vehicles, trade partners, and commodities, thus providing key information for pathway analysis and risk assessment [13]. For example, Ward et al. [10] found that the majority of ant species intercepted at New Zealand borders arrived from Pacific regions via fresh produce trade. More recently, Yang et al. [14] documented that most exotic ants intercepted at Gaoming port, Guandong Province, China, originated from Southeast Asian countries, with fruits serving as a predominant commodity type associated with the intercepted ants.

While more than 20 introduced ant species have been reported in Taiwan (including some globally distributed species such as the red imported fire ant, *Solenopsis invicta*, and the big-headed ant, *Pheidole megacephala* [15]), records concerning the interception of non-native ants at Taiwanese borders have been largely lacking. As of the year of 2011, identification of border-intercepted insects to the species level (when possible) was enforced, and records with information such as taxonomy, country of origin, and vector type were archived in a database, namely, the “Quarantine Information System”, established by the Bureau of Animal and Plant Health Inspection and Quarantine (BAPHIQ), a responsible authority in Taiwan for quarantine services of animal- and plant-associated pests and diseases. Such a database therefore provides a promising opportunity to render possible the identification of high-risk pathways for non-native ants into Taiwan.

The first objective of this study is to understand taxonomic patterns and common pathways of intercepted ants at the Taiwanese borders. We obtained all intercepted ant records from 2011 to 2018 in the database and analyzed the country of origin, type of commodity, and taxonomic diversity of these ant species, with a specific goal of identifying what species have been intercepted with high frequency and what commerce is most associated with the intercepted ants.

The second objective is to employ the dataset to answer three core questions related to ant invasion success. We note that invasion success is often investigated in a stage-dependent manner [16] as mechanisms underlying success at each stage can be substantially different [17]. Therefore, we examined success/risk explicitly as “establishment” success/risk to avoid potential confusion. First, does border interception data match the risk of establishment of exotic ants? We address this question by comparing the taxonomic composition of intercepted ants to introduced species already established in Taiwan. Such a comparison allows us to ask whether interception data alone can be used to predict the risk of establishment [18,19,20]. Second, are specific biological characteristics correlated with the establishment of exotic ants? Determinants of establishment success generally include propagule pressure (referring to both the number of individuals introduced and the number of introduction events [21,22,23], biotic (e.g., traits such as adaptation to human-modified conditions [8,9,10]), and abiotic factors (e.g., habitat suitability and/or characteristics of invaded habitats [24,25,26]). We focus on biotic factors and test whether species traits function as predictors for future ant invasion by comparing life-history traits between intercepted and established ant species in Taiwan. Third, are non-native ants intercepted at the Taiwanese borders primarily arriving from other introduced populations (i.e., secondary introductions) rather than from their native range (i.e., primary introductions)? There is increasing evidence that propagules of exotic species primarily originate from previously established populations rather than from their native ranges [27,28]. We therefore assess the prevalence of these secondary introductions in our interception dataset.

## 2. Materials and Methods

### 2.1. Analysis of Interception Records

Cargo, goods, and parcels arriving at the Taiwanese borders via air and sea transport routes were visually inspected by quarantine personnel on a random basis with a 5% inspection rate regardless of country origin or commodity type (https://www.baphiq.gov.tw/ws.php?id=3620). If ants were detected, the samples were collected, preserved in ethanol, and sent to the Social Insect Laboratory at National Changhua University of Education (NCUE) for identification and databasing. We obtained records of intercepted ants from the Quarantine Information System (BAPHIQ, Taiwan) for a period of 2011–2018. The interception database includes taxonomic information (species-level when possible, commodity information, and country of origin for every ant detected. Identification was based on both authoritative databases (i.e., AntWeb (www.antweb.org) or AntWiki (www.antwiki.org)) and the published literature [15,29]. We also used AntCat (https://www.antcat.org/), which contains 13,738 valid species names based on the Bolton World Catalog [30], to check species names for synonyms. Note that only those records identified to the species level were subjected to subsequent analyses, and genus-only records were summarized in Figure S1.

Commodities associated with the interception records were placed into the following categories: (1) log/timber, (2) wood products, (3) live plants (including fruits/vegetables), (4) bamboo/bamboo products, and (5) waste. The most frequently intercepted ant species and the main commodity on which ant species arrived were identified only in countries with more than 30 interception records to avoid potential bias introduced by a small number of interceptions.

### 2.2. Border Interception Record vs. Establishment Risk

To answer whether interception data matches establishment risk, taxonomic composition at the subfamily level between interception records and all established exotic ant species in Taiwan was compared and analyzed using a chi-squared goodness-of-fit test performed in SPSS version 16.0 (SPSS, Chicago, IL, USA). We used the subfamily level in the analysis because neither the genus nor species level would provide sample sizes with statistical robustness [10].

### 2.3. Predictors of Establishment Risk 

To identify major determinant(s) of establishment success of ants in Taiwan, two separate tests were carried out. The first test concerned whether worker size is associated with establishment success. We tested the significance of difference in body length (mm) between “ants intercepted but not established” and “intercepted ants that are established in Taiwan” using Student’s *t*-test (at a 95% confidence interval) performed in SPSS version 16.0 (SPSS, Chicago, IL, USA). The maximum body length of workers for these ant species was obtained from previously published data (Table S1).

The second test examined the effect of interception frequency and life-history trait on establishment risks using a generalized linear model with binomial error. We first assigned the following life-history traits to each species when possible: (1) queen number (monogyne, polygyne, or both); (2) colony founding mode (independent founding, dependent founding, or both); (3) nesting site preferences (general nesting habits, soil/litter, or arboreal nests/nesting inside wood; (4) body size (maximum body length of workers obtained from previously published data) (Table S1). Establishment risk was considered as a response variable and represented by 1 (established) and 0 (non-established) for each ant species. The effect of the life-history traits (i.e., colony founding mode, nesting site, queen number, worker size) and interception frequency were examined by selecting the model with subset combinations of the fixed effect terms according to the corresponding Bayesian information criterion (BIC) values and weight (wi). The analysis was conducted by the basic function and MuMIn packages in R [31]. Note that ant species native to Taiwan were excluded from the establishment analysis since our objective was to determine whether certain ant life-history traits and the number of interceptions contribute to their success in introduced regions. Establishment risk associated with each life-history trait (except worker size) was estimated by dividing the number of established ant species with a given life-history trait by the total number of intercepted species with the same trait (0 = lowest risk; 1 = highest risk).

### 2.4. Prevalence of Secondary Introductions

We used the AntMaps database [32] to assess native and exotic ranges of all intercepted ant species. For exotic species intercepted at the borders, we estimated the percentage of primary introductions (intercepted cargo originating from species’ native ranges) and secondary introductions (intercepted cargo originating from species’ invaded ranges). We also tested whether there is a significant difference in the percentage of secondary introductions between “exotic” and “invasive” species. Note that we defined a subset of exotic species as invasive species based on the criteria of the International Union for the Conservation of Nature (http://www.iucngisd.org/gisd/) (e.g., if species pose negative effects on biodiversity, agriculture, health, and/or ecosystem functioning). Significance was determined using Pearson’s chi-square test at a 95% confidence interval.

## 3. Results

### 3.1. Analysis of Interception Records

Over a period of eight years (2011 to 2018), there were 461 BAPHIQ records of ant interception at the Taiwanese borders. A majority of ant sample derived from maritime cargo (439, more than 95% of the total interceptions), while the remaining ant samples derived from plants or other products carried by passengers in the airport and/or by mail. Subsequent analyses were thus carried out only on the maritime-cargo-associated interception data. Of all the maritime-cargo-associated records, 236 (53.8%) were identified to the species level and the rest to the genus level. A total of 52 ant species from 24 genera were intercepted at the borders (Table 1). Worker was the predominant caste, as more than 97% of the intercepted samples comprised workers only. Fifteen species had more than five records, 20 species had two to five records, and 17 species were recorded only once in the database. The most frequently intercepted species was *Crematogaster teranishii,* which contributed to 9.7% of all records, followed by *Camponotus kiusiuensis* and *Cr*. *matsumurai* (Table 1). Thirteen of the intercepted species (marked with an asterisk in Table 1) are considered invasive ants in Taiwan. Among ants only identified to the genus level, the genera *Camponotus*, *Crematogaster,* and *Tetraponera* jointly contributed 49.3% of all records (Figure S1).

**Figure 1:**
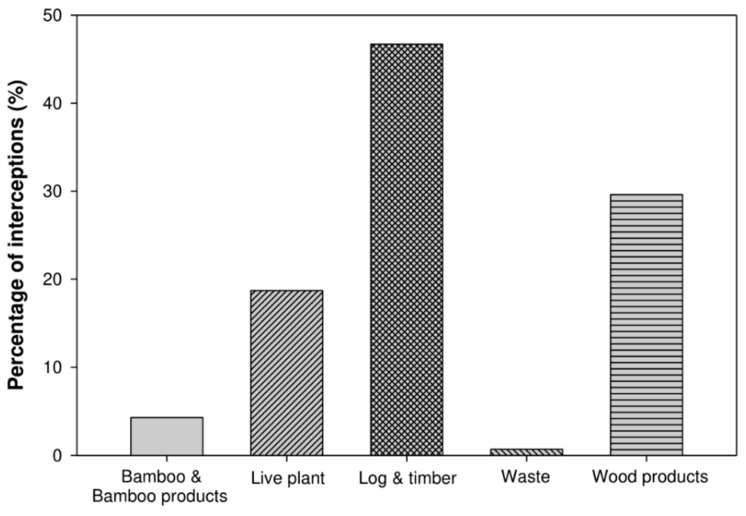
Type of commodity with which intercepted ants were associated from 2011 to 2018.

Intercepted ants originated from 31 different countries, with Vietnam being the most common (contributing 29.2% of all interception records), followed by Japan, China, USA, Thailand, and Laos. Analyses of the proportion of interception by country across 2011–2018 revealed that the top three countries during 2011–2018 are, coincidentally, either Taiwan’s top trading partners or top forest-product-importing countries (http://www.trade.gov.tw/) (Table S2). This pattern may reflect differences in the trade volume of each country with Taiwan, since quarantine inspections were conducted randomly. The most common commodity type on which ant species arrived into Taiwan was log/timber (46.7%; Figure 1). However, the predominant commodity on which stowaways arrived varied by country of origin (Table 2). For both Japan and the USA, log/timber was the most prevalent vector of exotic ants, whereas exotic ants from China arrived predominantly on live plants. Similarly, ant species intercepted most frequently also varied among countries. For example, *Cr*. *teranishii* and the ghost ant *Tapinoma melanocephalum* were the most frequently intercepted species from Japan and Thailand, respectively.

### 3.2. Border Interception Record vs. Establishment Risk

Five subfamilies were represented in the intercepted records (Table 3). The number of intercepted ant species at the borders differed significantly with current established exotic ant species in Taiwan (χ^2^ = 38.383, d.f. = 2, *p* < 0.01), with proportionally more Formicinae and yet fewer Myrmicinae in the interception records.

### 3.3. Predictors of Establishment Risk 

Using a generalized linear model, we found that the best model explaining the ant establishment risk contains the colony founding mode and the number of interception records as fixed effects, whereas queen number did not appear in the first six models ranked by BIC (Table 4). Nevertheless, the number of interception records was included in most of the first ten selected models. Thus, our data suggest that ant species with higher interception frequencies and mixed colony founding modes (independent and dependent) are more likely to become established. In contrast, queen number was unlikely to affect establishment success. However, it is worth mentioning that the establishment risk for strictly monogyne species was 0.0 ± 0.0 (Figure S2). Specifically, propagule pressure and a selected suite of life-history traits (i.e., colony founding mode) were associated with the probability of ant establishment. For example, among those intercepted ants that are established, a majority of them are able to initiate a new colony both independently and dependently (66.7%). Moreover, the establishment risk for those species with two colony founding modes is 1.0 ± 0.0 (Figure S2).

We, however, could not rule out nesting site and worker size as potential determinants of the establishment risk of ant species as both traits appeared in the second ranked model. Ant species with general nesting site requirements had a relatively higher establishment risk (i.e., 0.7 ± 0.2), whereas arboreal ant species had a relatively lower establishment risk (i.e., 0.1 ± 0.1) (Figure S2). Hence, it appears that nesting site was likely associated with the probability of ant establishment. Mean body length of established ants (3.9 ± 0.6 mm) tended to be smaller than ants that were intercepted but not known to be established (7.6 ± 1.2 mm), but not significantly different (*t* = 2.034, d.f. = 26, *P* = 0.052). Thus, worker size may be associated with the probability of ant establishment in introduced regions.

### 3.4. Prevalence of Secondary Introductions

Regarding the prevalence of secondary introduction, more than half of interception records of introduced species arrived into Taiwan from their native ranges (64.3%) (Table S4). However, when analyzing “exotic” and “invasive” species separately, we found that the proportion of secondary introductions for invasive species was higher (70.8%) than for exotic species (14.8%) (χ^2^ = 41.223, d.f. = 1, *p* < 0.01).

## 4. Discussion

### 4.1. Overrepresentation of Arboreal Ants

The majority of exotic ants arrived on log/timber, likely representing a consequence of the forestry policy reform in Taiwan. Multiple practices to preserve natural environments, including a ban on logging, were implemented in Taiwan since 1991. Virtually all wood or timber products (≈99%) are imported from overseas to fulfill domestic demand [33,34]. According to import records of forest products between 2003 and 2013 compiled by the Taiwan Forestry Research Institute, the average value of roundwood imported into this region is up to approximately US $200 million [35]. Such a pattern corroborates the prediction that higher trade volume (forest products in this case) likely leads to a higher number of interceptions of exotic species (ants, in this case) [36].

The three most frequently intercepted species, *Cr*. *teranishii*, *C*. *kiusiuensis,* and *Cr*. *matsumurai* (collectively contributed 23.3% of all records), are characterized as arboreal ants. A similar pattern was found at the genus level, in that ant species belonging to the genus *Camponotus*, *Crematogaster,* and *Tetraponera* are the most frequently intercepted taxa at the borders (Figure S1). Many *Crematogaster* species are arboreal, nesting in decayed parts of standing trees, branches, or twigs on trees, with some species also building carton nests [37,38,39], whereas *Camponotus* species are known as one of the most common ants that typically nest in the wood of rotting logs, under bark, and in decayed parts of live or dead trees [40,41]. Similarly, *Tetraponera* species are arboreal nesters, nesting in rotting logs, cavities of living plants, and branches of trees [42]. Given the high rate of interception of *Cr*. *teranishii*, it is surprising that no established population is known in Taiwan or elsewhere. One plausible explanation is that the interception record in this study is rather recent (2011 to 2018), and it may have already established in Taiwan but remains undetected due to a low population density (e.g., a lag phase) [43,44] and/or localized population [45]. Another possibility for such asymmetry may involve other factors such as biological characteristics or ecological attributes (see Section 4.2 for more details). Interestingly, some of these frequently intercepted arboreal species (e.g., *C*. *kiusiuensis* and *Cr*. *matsumurai*) are also native to Taiwan, raising a potential concern that these species could spread to areas where geographically isolated populations of these ants are enabled to come into contact and thus gene exchange between distinct gene pools may occur. Although the consequences of such “man-made” contact remain to be determined, a number of recurrent pest outbreaks and control failures have been associated with population admixture as a result of hybridization of genetically distinct sources [46,47].

### 4.2. Predictors of Establishment Risk

Propagule pressure is thought to function as a major determinant for both establishment success and the subsequent spread of exotic species [21,22,48]. The greater number of individuals released or of introduction events, the higher the probability that a population will become established. However, our analyses suggest that the invasion success of exotic ant species may not only depend on propagule pressure alone. For example, some invasive ants that are established in Taiwan did not have high interception frequencies (e.g., red imported fire ant, *S*. *invicta*: four interceptions; tropical fire ant, *S*. *geminata*: 14 interceptions; *Ta*. *melanocephalum*: 14 interceptions). However, one should interpret these data with caution as quarantine effort or detection methodology itself may influence the number of interceptions of these species. A more extreme example is *Cr. teranishii*, which has been frequently intercepted at the Taiwanese borders but has not yet been detected or established locally.

Invasive ants tend to share a suite of traits that have been believed to increase their probability of establishment in new regions [8,12,49,50], and these traits include general nesting and habitat requirements, relatively small worker body size, polygyny, budding colony founding, and the tendency to form supercolonies. Our analyses, however, suggest that only a subset of these life-history traits (i.e., colony founding mode, nesting site and perhaps body size) play a role in the establishment success of ants in Taiwan (Table 4). Consistent with this prediction, the invasive fire ant *S*. *invicta* has been confirmed to have established multiple persistent populations that are currently expanding in Taiwan, and its establishment success has been linked to flexibility in its colony founding mode [14]. Arboreal ants often possess a specific preference for nesting sites that may not be abundant in port areas. For example, *Cr*. *matsumurai* nest in decayed sections of relatively tall trees [39] and are unlikely to establish populations in locations without adequate forest cover. In contrast, numerous tramp/invasive ants prefer to nest in open or disturbed habitats and, therefore, are more likely to establish populations in port areas (e.g., in containers, within container yards) [51,52]. We therefore argue that an interaction between propagule pressure and biological traits such as colony founding mode (and possibly other factors such as habitat suitability) may be required for establishment success of introduced ants.

Ants are often stowaways and/or hitchhikers in cargo containers rather than being associated with a particular commodity [2,53]. Our database, however, is in favor of commodity type serving as a general predictor of intercepted ant species, at least at a functional group level. For example, one could predict that arboreal and/or wood-dwelling ants are more likely to be intercepted in wood products and log/timber. One additional example is derived from the analysis of interception records in Thailand, in which more than 96% of ants were found to be associated with live plants, with the species *Ta*. *melanocephalum*, *Technomyrmex brunneus,* and *Tec*. *horni* collectively accounting for 75% of the intercepted records. These ants are either species commonly associated with live plants or mutualistically associated with honeydew producers that feed on live plants [54,55,56,57].

### 4.3. Secondary Introductions

Most interception records for introduced ant species at the Taiwanese borders originated from populations within the species’ native ranges (64.3%). The proportion of primary introductions in our study appears to be higher than what was reported in the USA and New Zealand datasets (24.3% and 12.2% for USA and New Zealand, respectively) [28]. This may be largely due to the over-representation of arboreal ant species among exotic ant species intercepted in our database (50%), and arboreal ants are rarely reported to establish introduced populations elsewhere. Nevertheless, consistent with Bertelsmeier et al. [28], our analyses also show that most of the invasive ant species arrived in Taiwan via secondary introductions, possibly due to the disproportionate abundance of invasive ants in non-native regions. Many invasive ants are ecologically dominant species with high population densities in their introduced ranges [49,58]. Combined with several traits that favor their adaptation to human-modified environments and conditions [12,49], the likelihood of being transported by human and/or surviving during a voyage appears to be high, thus promoting secondary introduction as a major driver of global invasions of ants.

### 4.4. Quarantine Implications

The current study provides practical information for the implementation of early detection of invasive ant species. For example, the BAPHIQ can prioritize the screening of shipments based on predictors proposed in this study. It is worth mentioning that all of the five worst invasive ant species (listed as the world’s top 100 invasive species by the IUCN; [59]) were detected in our interception records, although most at low interception frequencies (e.g., the Argentine ant, *Linepithema humile,* and little fire ant, *Wasmannia auropunctata*, were each intercepted once in a period of 8 years). Previous studies have shown that establishment of invasive species may occur even with limited interception records [50,60]. Hence, apart from prioritization of the predominant trade pathways (i.e., country of origin) and commodities with which exotic ants were intercepted, quarantine authorities should also emphasize tracing and monitoring the trade pathways with which these most unwanted non-native ants are associated. To target those invasive species, quarantine authorities should also prioritize the inspection of cargo originating from secondary introductions instead of just focusing on cargo originating from their native range. Furthermore, tracking the post-border movement of associated commodities as well as monitoring commodity storage sites would ensure an early detection and prompt notification of the presence of these species if they have been established.

## 5. Conclusions

Our study show that border interception data alone may not reveal the establishment risk of introduced ants, but a framework integrating both propagule pressure and species traits (i.e., flexible colony founding mode and general nesting requirements) may provide a better resolution for predicting future ant invasions. Furthermore, most invasive ants arrived in Taiwan via secondary introductions, a pattern seen in other biogeographic regions including Oceania (New Zealand, Bertelsmeier et al. [28], and Australia, Suhr et al. [61]) and the USA (Bertelsmeier et al. [28]). An effective quarantine strategy in response to recurrent secondary introductions at a global scale must incorporate a thorough knowledge of introduced populations for species of interest. Lastly, our results suggest that countries with a high demand for timber imports may risk receiving non-native arboreal ants or ant species showing an affinity for this commodity type. Whether these ants will represent a new biosecurity issue remains to be seen; however, phytosanitary measures specifically targeting ants associated with log/timber should be developed and implemented in the near future.

## Figures and Tables

**Table 1:** List of ant species intercepted at the Taiwanese borders based on the database “Quarantine Information System” established by the Bureau of Animal and Plant Health Inspection and Quarantine (BAPHIQ) from 2011 to 2018.

Species ^a^	Number of Records	Established Status ^b,c^
More than five interceptions		
* Crematogaster teranishii*	23	–
* Camponotus kiusiuensis*	17	Ea/Na
* Crematogaster matsumurai*	15	Na
Solenopsis geminata *	14	Ex
Tapinoma melanocephalum *	14	Na/Tr/Ws
Paratrechina longicornis *	12	Ex/Tr/Tw
* Tetramorium nipponense*	10	Ea/Na
* Tetraponera nigra*	10	–
Anoplolepis gracilipes *	9	Ex/Tw
* Pristomyrmex punctatus*	9	IA/Na/Or
* Vollenhovia emeryi*	8	Ea
* Technomyrmex brunneus*	7	Ea/Na/Tw
* Camponotus pennsylvanicus*	7	–
* Dolichoderus thoracicus*	6	IA/Na/Or
* Technomyrmex gibbosus*	6	–
Two–five interceptions		
Brachyponera chinensis *	5	Na/Or
* Camponotus herculeanus*	5	–
Solenopsis invicta *	4	Ex
* Tetramorium pacificum*	4	Na/Tr
Monomorium pharaonis *	3	Ex/Tr
* Camponotus keihitoi*	3	–
Pheidole megacephala *	2	Ex
* Pheidole nodus*	2	Na/Or
Technomyrmex albipes *	2	Ea/Na/Tw
* Technomyrmex horni*	2	Na/Tw
* Tetramorium simillimum*	2	Ex/Tr
Trichomyrmex destructor *	2	Ex
* Camponotus bishamon*	2	–
* Camponotus hemichlaena*	2	–
* Camponotus nawai*	2	Na
* Camponotus obscuripes*	2	–
* Lasius japonicus*	2	Ea/Na
* Lasius productus*	2	–
* Lasius sakagamii*	2	–
* Temnothorax makora*	2	–
One interception		
* Camponotus variegatus dulcis*	1	Na/Or
* Crematogaster dohrni fabricans*	1	IA/Na/Or
* Monomorium intrudens*	1	Na/Tr
Monomorium floricola *	1	Na/Tr
* Brachyponera luteipes*	1	Ea/Na
* Nylanderia amia*	1	Ea/Na
* Pheidole fervens*	1	Na/Or
* Polyrhachis illaudata*	1	IA/Na/Or
* Tetramorium lanuginosum*	1	Na/Or
* Camponotus novaeboracensis*	1	–
* Camponotus singularis*	1	–
* Crematogaster egidyi*	1	–
* Formica japonica*	1	Na/Or
Linepithema humile *	1	–
* Oecophylla smaragdina*	1	–
* Tetramorium caespitum*	1	–
Wasmannia auropunctata *	1	–

^a^ Ant species that are classified as invasive by the International Union for the Conservation of Nature (IUCN) are denoted by an asterisk (*). ^b^ For those ant species with known established populations in Taiwan, the distribution or status of the ant species is indicated as: Ea = East-Asia-distributed species; Na = native species; Ex = known exotic species; IA = Indo-Australasian species; Or = Oriental species; Tr = known tramp species; Tw: Taiwan-wide-distributed species; Ws: Worldwide-spread species. ^c^ AntWiki was used for the definition of biogeographic regions (https://antwiki.org/wiki/Category:Biogeographic_Region).

**Table 2:** Top five countries of origin ranked by number of ant interceptions, and the primary commodity type and ant species for each respective country.

Country	Number of Records	Primary Commodities (% of Records)	Primary Species (% of Records)
Vietnam	128	Wood products (75) Log/timber (23.4)	*Tetraponera nigra* (24.4) *Paratrechina longicornis* (19.5) *Tetramorium nipponense* (14.6)
Japan	121	Log/timber (84.3) Wood products (15.7)	*Crematogaster teranishii* (21.3) *Camponotus kiusiuensis* (15.7) *Crematogaster matsumurai* (12)
China	43	Live plants (46.5) Bamboo/Bamboo products (44.2)	*Tapinoma melanocephalum* (22.2) *Dolichoderus thoracicus* (16.7) *Technomyrmex brunneus* (16.7)
USA	35	Log/timber (91.4) Live plants (5.7)	*Camponotus pennsylvanicus* (41.2) *Camponotus herculeanus* (23.5) *Solenopsis geminata* (23.5)
Thailand	32	Live plants (96.9) Log/timber (3.1)	*Tapinoma melanocephalum* (50) *Technomyrmex brunneus* (12.5) *Technomyrmex horni* (12.5)

**Table 3:** The number of ant species from different subfamilies intercepted by BAPHIQ and the number of exotic ant species established in Taiwan.

Subfamily	Interception Records (%)	Intercepted/Established ^a^ (%)	Exotic/Established ^b^ (%)
Dolichoderinae	7 (13.5)	0	1 (6.7)
Formicinae	20 (38.5)	2 (22.2)	3 (20.0)
Myrmicinae	22 (42.3)	7 (77.8)	10 (66.7)
Ponerinae	2 (3.8)	0	1 (6.7)
Pseudomyrmecinae	1 (1.9)	0	0

^a^ Intercepted exotic species with known established populations in Taiwan. ^b^ Exotic ant species with known established populations in Taiwan; see Table S3.

**Table 4:** The top ten models set with corresponding BIC values and rounded model weights used to assess the effect of life-history traits and number of interception records on the establishment risks of ant species.

Rank	(Intercept)	Fixed Effect ^a^					*R* ^2^	df	BIC	Weight
		C.F.M.	I.R.	W.S.	N.S.	Q.N.				
1	−4.10 × 10^1^	+ ^a^	20.49				0.69	4	16.10	0.29
2	−2.38 × 10^3^		108.80	−35.27	+		0.72	5	16.66	0.22
3	−9.35 × 10^2^		39.65		+		0.67	4	17.15	0.17
4	4.61 × 10^−21^	+					0.62	3	18.31	0.10
5	−4.25 × 10^1^	+	20.17	0.61			0.69	5	19.43	0.06
6	−9.23 × 10^2^	+	39.12		+		0.72	6	19.99	0.04
7	−4.24 × 10^1^		56.45	−19.95		+	0.67	5	20.48	0.03
8	−1.76 × 10^0^	+		0.65			0.62	4	21.36	0.02
9	−4.05 × 10^1^	+	20.25			+	0.69	6	22.77	0.01
10	−7.12 × 10^2^		38.96		+	+	0.69	6	22.77	0.01

C.F.M., colony founding mode; I.R., interception records; W.S., worker size; N.S, nesting size; Q.N., queen number. ^a^ “+” indicated that the respective life-history trait was considered as a fixed factor in the model.

## Supplementary Materials

The following are available online at https://www.mdpi.com/2075-4450/11/6/356/s1, Figure S1: Ants intercepted at the Taiwanese borders from 2011 to 2018 that can only be identified to the genus level, Figure S2: Establishment risk of intercepted ants with different life-history traits. (**a**) Colony founding mode; (**b**) Nesting site; (**c**) Queen number, Table S1: Life-history traits and worker size of ant species intercepted at the Taiwanese borders, Table S2: Proportion of interceptions by country through the years, focused on the top three countries of origin for intercepted ant species, Table S3: List of exotic ant species with known established populations in Taiwan, Table S4: Proportion of primary introductions and secondary introductions of non-native species that are intercepted at Taiwan borders.
