# Supplementary material for: Analysis of Recent Interception Records Reveals Frequent Transport of Arboreal Ants and Potential Predictors for Ant Invasion in Taiwan
Source: Insects. 2020 Jun 8;11(6):356. doi: 10.3390/insects11060356 (PMC7349007; doi:10.3390/insects11060356)

Supplementary Materials

Analysis of Recent Interception Records Reveals Frequent Transport of Arboreal Ants and Potential Predictors for Ant Invasion in Taiwan

Ching-Chen Lee, Yi-Ming Weng, Li-Chuan Lai, Andrew Suarez, Wen-Jer Wu, Chung-Chi Lin, and Chin-Cheng Scotty Yang

Table S1 Life-history traits and worker size of ant species intercepted at the Taiwanese borders

Ant species	Queen number			Colony founding mode			Nesting site			Total body length (max)
	Monogyne	Polygyne	Both	Independent	Dependent	Both	General	Soil	Arboreal	
**Exotic species that are intercepted but not established**										
Camponotus bishamon	√			√					√	5.0 mm
Camponotus hemichlaena	√			√					√	12.0 mm
Camponotus herculeanus		√		√					√	13.0 mm
Camponotus keihitoi	√			√					√	4.5 mm
Camponotus novaeboracensis	√			√					√	12.0 mm
Camponotus obscuripes.	√			√					√	12.0 mm
Camponotus pennsylvanicus	√			√					√	14.0 mm
Camponotus singularis	√			√				√		19.0 mm
Crematogaster egidyi	√			√					√	4.0 mm
Crematogaster teranishii	√			√					√	4.0 mm
Lasius productus	√			√				√		4.5 mm
Lasius sakagamii		√			√			√		3.5 mm
Linepithema humile		√			√		√			2.5 mm
Oecophylla smaragdina	√			√					√	11.0 mm
Technomyrmex gibbosus	√			√					√	2.5 mm
Temnothorax makora			√	√					√	2.2 mm
Tetramorium caespitum	√			√				√		4.0 mm
Tetraponera nigra	√			√					√	13.0 mm
Wasmannia auropunctata			√		√		√			1.5 mm
**Exotic species that are intercepted & established**										
Anoplolepis gracilipes		√				√	√			4.0 mm
Monomorium pharaonis		√			√		√			2.4 mm
Paratrechina longicornis		√			√		√			2.5 mm
Pheidole megacephala		√			√		√			3.5 mm
Solenopsis geminata			√			√		√		8.0 mm
Solenopsis invicta			√			√		√		6.0 mm
Tetramorium simillimum		√				√		√		2.5 mm
Trichomyrmex destructor		√				√	√			3.5 mm
Vollenhovia emeryi			√			√			√	2.5 mm

Ant species	Queen number			Colony founding mode			Nesting site			Total body length (max)
	Monogyne	Polygyne	Both	Independent	Dependent	Both	General	Soil	Arboreal	
**Native species that are intercepted**										
Brachyponera chinensis			√	√				√		3.5 mm
Brachyponera luteipes		√		√				√		3.5 mm
Camponotus kiusiuensis	√			√					√	11.0 mm
Camponotus nawai			√			√			√	4.5 mm
Camponotus variegatus dulcis	√			√					√	12.5 mm
Crematogaster dohrni fabricans	√			√					√	4.0 mm
Crematogaster matsumurai			√	√					√	3.5 mm
Dolichoderus thoracicus		√				√	√			4.5 mm
Formica japonica			√			√		√		6.0 mm
Lasius japonicus	√			√				√		3.5 mm
Monomorium intrudens		√			√				√	1.5 mm
Monomorium floricola		√			√		√			2.0 mm
Nylanderia amia		√			√		√			3.0 mm
Pheidole fervens		√			√			√		4.5 mm
Pheidole nodus		√			√			√		3.0 mm
Polyrhachis illaudata	√			√					√	12.0 mm
Pristomyrmex punctatus[a]				√				√		2.5 mm
Tapinoma melanocephalum		√			√		√			2.0 mm
Technomyrmex albipes		√				√			√	2.5 mm
Technomyrmex brunneus		√				√		√		2.8 mm
Technomyrmex horni		√		√				√		4.3 mm
Tetramorium pacificum		√		√				√		4.6 mm
Tetramorium lanuginosum	√			√				√		2.3 mm
Tetramorium nipponense		√		√					√	3.7 mm

[a] *Pristomyrmex punctatus* has no queens and reproduces parthenogenetically

Table S2 Proportion of interception by country from 2011 to 2018, focused on top three countries of origin for intercepted ant species

Year	Primary country of origin for ant interceptions (% of interceptions)
2011	Vietnam (66.7%)<br>China (20.0%)<br>Japan (6.7%)
2012	Vietnam (42.1%)<br>China (28.9%)<br>Japan (18.4%)
2013	Japan (34.4%)<br>China (28.1%)<br>Vietnam (12.5%)
2014	China (25.8%)<br>Japan (22.6%)<br>Laos (22.6%)
2015	Japan (45.5%)<br>Vietnam (18.2%)<br>USA (9.1%)
2016	Japan (57.1%)<br>Thailand (10.4%)<br>Vietnam (9.1%)
2017	Vietnam (47.7%)<br>Japan (14.8%)<br>USA (10.2%)
2018	Vietnam (38.8%)<br>Thailand (18.4%)<br>Japan (12.6%)

Table S3 List of exotic ant species with known established populations in Taiwan

Subfamily	Species
Dolichoderinae	*Dolichoderus thoracicus*
Formicinae	*Anoplolepis gracilipes*
	Paratrechina longicornis
	Plagiolepis alluaudi
Myrmicinae	*Monomorium pharaonis*
	Pheidole megacephala
	Solenopsis geminata
	Solenopsis invicta
	Tetramorium simillimum
	Trichomyrmex destructor
	Vollenhovia emeryi
	Strumigenys emmae
	Strumigenys membranifera
	Strumigenys rogeri
Ponerinae	*Hypoponera ergatandria*

Table S4 Proportion of primary introductions and secondary introductions of non-native species that are intercepted at Taiwanese borders

Species	Status [a]	Total number of interceptions	Native regions	Proportion of interceptions originated from species' native region	List of regions or countries that contribute to secondary introductions [b]
Anoplolepis gracilipes	Invasive	9	Indo-Australian, Oriental	100%	–
Camponotus bishamon	Exotic	2	Japan	100%	–
Camponotus hemichlaena	Exotic	2	Japan	100%	–
Camponotus herculeanus	Exotic	5	Palaearctic, Nearctic	100%	–
Camponotus keihitoi	Exotic	3	China, Japan, South Korea	100%	–
Camponotus novaeboracensis	Exotic	1	Canada, USA	100%	–
Camponotus obscuripes	Exotic	2	Japan	100%	–
Camponotus pennsylvanicus	Exotic	7	Canada, USA	100%	–
Camponotus singularis	Exotic	1	Indo-Australian, Oriental, Palaearctic	100%	–
Crematogaster egidyi	Exotic	1	Indo-Australian, Palaearctic	100%	–
Crematogaster teranishii	Exotic	23	Japan, Korea	100%	–
Lasius productus	Exotic	2	Japan, Korea	100%	–
Lasius sakagamii	Exotic	2	Japan	100%	–

Linepithema humile	Invasive	1	Neotropical	0%	Netherlands (100%)
Monomorium pharaonis	Invasive	3	Neotropical	0%	Australia (33.3%), Vietnam (66.7%)
Oecophylla smaragdina	Exotic	1	Australasian, Indo-Australian, Oriental, Palaearctic	100%	–
Paratrechina longicornis	Invasive	12	Neotropical	0%	Australia (16.7%), Canada (8.3%), Vietnam (66.7%), Philippines (8.3%)
Pheidole megacephala	Invasive	2	Neotropical	0%	Vietnam (100%)
Solenopsis geminata	Invasive	14	Neotropical, southeast USA	35.7%	Australia (7.1%), Canada (28.6%), China (7.1%), Vietnam (21.4%)
Solenopsis invicta	Invasive	4	Neotropical	0%	Canada (75%), Japan (25%)
Technomyrmex gibbosus	Exotic	6	Japan, Korea	100%	–
Temnothorax makora	Exotic	2	Japan	100%	–
Tetramorium caespitum	Exotic	1	Palaearctic	100%	–
Tetramorium simillimum	Exotic	2	Afrotropical	0%	Philippines (50%), Vietnam (50%)
Tetraponera nigra	Exotic	10	Indo-Australian, Oriental, Palaearctic	0%	Vietnam (100%)
Trichomyrmex destructor	Invasive	2	Oriental	50%	UK (50%)
Vollenhovia emeryi	Exotic	8	Japan, Korea	100%	–
Wasmannia auropunctata	Invasive	1	Neotropical	0%	Solomon Island (100%)

[a] Exotic species referred to ant species that is not native Taiwan; invasive species are subset of exotic species that posed negative effects on biodiversity, agriculture, health and/or ecosystem functioning (http://www.iucngisd.org/gisd/).

[b] Proportions of interceptions for each respective region or country are indicated in parentheses.

Figure S1 Intercepted ants that only can be identified to genus level at the Taiwanese borders from 2011 to 2018.

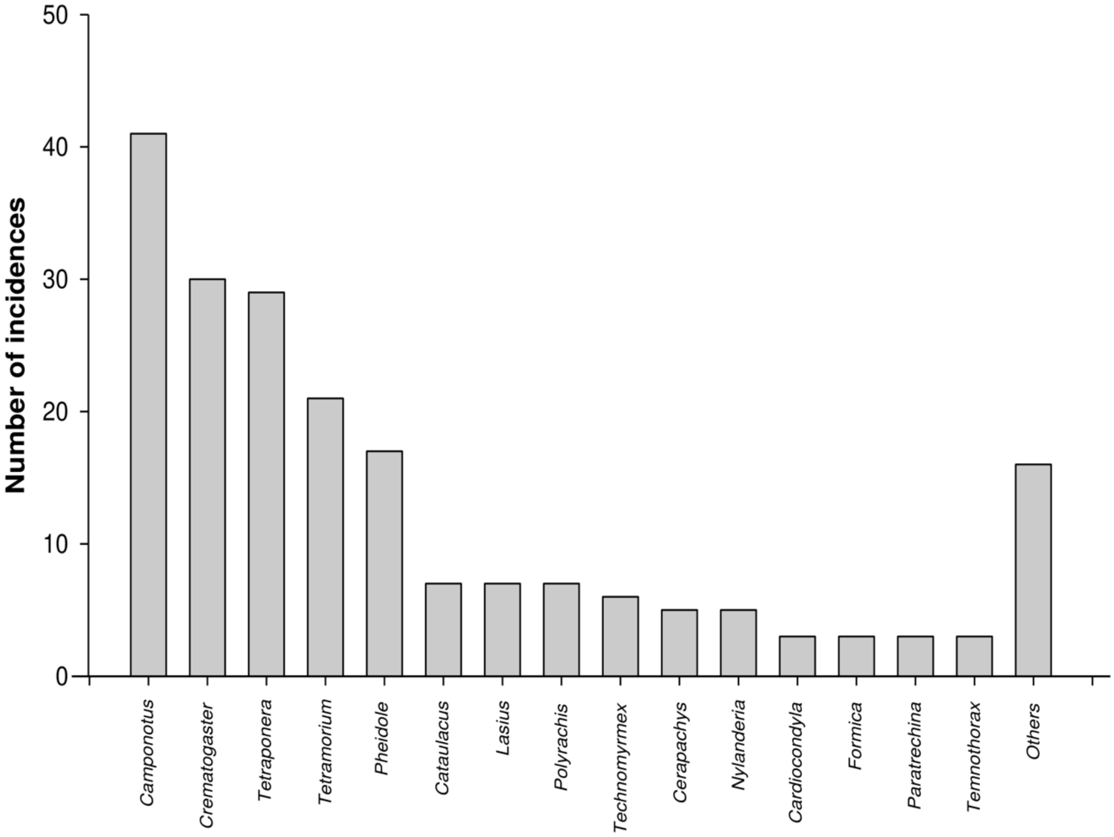

Figure S2 Establishment risk of intercepted ants with different life-history traits. (a) Colony founding mode (b) Nesting site (c) Queen number.

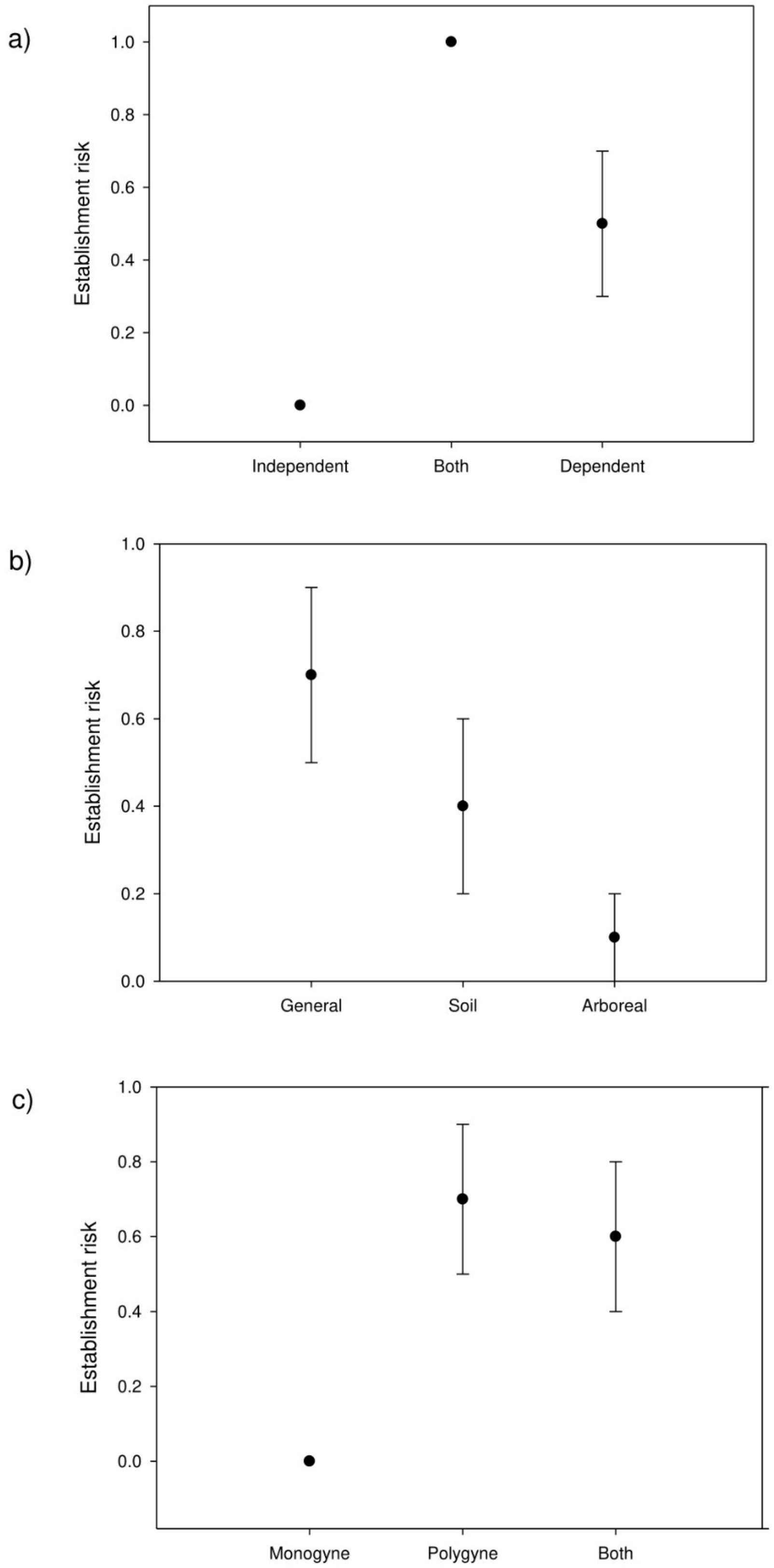

Supplement: Supplementary file 1 [file insects-11-00356-s001.pdf]
